# Comparing Occurrence of Bovine Respiratory Pathogens Detected by High-Throughput Real-Time PCR in Nasal Swabs and Non-Endoscopic Bronchoalveolar Lavage Samples from Dairy and Veal Calves

**DOI:** 10.3390/pathogens13060479

**Published:** 2024-06-05

**Authors:** Nina Dam Otten, Nicole Bakkegård Goecke, Anne Marie Michelsen, Liza Rosenbaum Nielsen, Nynne Capion, Henrik Læssøe Martin, Bodil Højlund Nielsen, Lars Erik Larsen, Mette Bisgaard Petersen

**Affiliations:** 1 Department of Veterinary and Animal Sciences, Faculty of Health and Medical Sciences, University of Copenhagen, Grønnegårdsvej 15, DK-1870 Frederiksberg, Denmark; nio@sund.ku.dk (N.D.O.); nbgo@sund.ku.dk (N.B.G.); amm@sund.ku.dk (A.M.M.); liza@sund.ku.dk (L.R.N.); lael@sund.ku.dk (L.E.L.); 2 Department of Veterinary Clinical Sciences, Faculty of Health and Medical Sciences, University of Copenhagen, Højbakkegårds Allé 5A, DK-2630 Taastrup, Denmark; nyc@sund.ku.dk; 3 SEGES Innovation, Agro Food Park 15, DK-8200 Aarhus, Denmark; hlm@seges.dk; 4 SimHerd A/S, Agro Business Park, Niels Pedersens Alle 2, DK-8830 Tjele, Denmark; bhn@simherd.com

**Keywords:** bovine, respiratory disease, pathogen, multiplex, PCR, nasal swab, bronchoalveolar lavage, comparison

## Abstract

This study aimed to enhance our understanding of the agreement between two sampling methods for the detection of bovine respiratory disease (BRD) pathogens in calves using high-throughput real-time qPCR (ht-RT-qPCR). In total, 233 paired nasal swab (NS) and non-endoscopic bronchoalveolar lavage (nBAL) samples were collected from 152 calves from 12 Danish cattle herds. In 202 of the observations, the calves were examined using a standardized clinical protocol. Samples were tested for three viruses (bovine respiratory syncytial virus, bovine corona virus, and influenza D virus) and six bacteria (*Histophilus somni*, *Mannheimia haemolytica*, *Mycoplasma bovis*, Mycoplasma species, *Pasteurella multocida*, and *Truepurella pyogenes*). The results showed age-related differences in disease and pathogen occurrence, with the highest detection rates in calves aged 35 days or older. Poor to moderate agreement was found between the NS and nBAL results. The presence of *Mannheimia haemolytica* in both NS and nBAL in younger calves and in nBAL in older calves was associated with clinical BRD. There was a potential link between BRD and influenza D virus in older calves, although it was only found in one herd in a small sample size. Overall, NS was a relatively poor predictor of pathogens in the lower respiratory tract. The present study confirms the complexity of pathogen detection in BRD, with marked influences of age and the sampling method on pathogen detection and disease associations.

## 1. Introduction

Bovine respiratory disease (BRD) is a highly prevalent disease complex that affects calves in all production systems, resulting in reduced animal welfare, undesirable antimicrobial use, and economic losses due to reduced productivity and increased mortality. BRD is a multifactorial disease complex associated with environmental factors, host factors, and multiple pathogens [1,2]. The wide range of viruses and bacteria involved in BRD includes, for example, bovine coronavirus (BCoV), bovine respiratory syncytial virus (BRSV), *Histophilus somni* (HS), *Mannheimia haemolytica* (MH), Mycoplasma species (M*spp.*), in particular *Mycoplasma bovis* (MB), and *Pasteurella multocida* (PM). In addition to these well-known BRD-causing pathogens, influenza D virus (IDV) and *Trueperella pyogenes* (TP) have also been associated with BRD [3,4,5].

Monitoring and testing diseased calves for pathogen involvement are important for choosing the appropriate treatment and can guide tailored vaccination programs. Different sampling techniques and analytical methods for detecting pathogens involved in BRD have been described, all of which have limitations [6]. Transtracheal aspirate (TTA) is often considered the best way to determine which pathogens are involved in a case of BRD, as the site of infection can be sampled without risk of contamination from the upper airways [6]. However, this technique is time-consuming and has potential welfare complications for calves, owing to the need for a surgical procedure [6]. For a method to be useful in a herd setting and to aid in increasing the knowledge about pathogens in cattle herds, it must be practically applicable, easy, affordable, and quick to perform. The obvious choices include sampling with a nasal swab (NS) and non-endoscopic bronchoalveolar lavage (nBAL), the latter being proposed as an increasingly used method [7]. NS is affordable and practical to use in the field, as swabbing young calves is relatively easy and quick compared with the more cumbersome and expensive technique of deep nasopharyngeal swabbing.

Knowledge of the limitations and the interpretation of the results are key elements before establishing the best sampling regimes. Doyle et al. [8] found good agreement between bacterial pathogens in NS and TTA, while the agreement was only moderate for the viral components of BRD. For MH, NS was predictive of the bacterial isolate found in TTA [9]. For MB, Pohjanvirta et al. [10] found that NS sampling was the most cost-effective method at the herd level. They used both real-time polymerase chain reaction (RT-PCR) and bacterial culture for pathogen detection. However, most older studies used only bacterial culture, which excludes viral pathogens from the analysis and leaves several days from sampling to the availability of the test results. PCR methods are becoming increasingly popular for pathogen detection because they are typically faster, more sensitive, and very specific. In recent years, multiplex RT-PCR tests targeting pathogens involved in BRD have been developed and used for the rapid identification of associated pathogens [11,12,13]. A limitation of multiplex RT-PCR is the number of targets that can be tested within a single run. Multiplex RT-qPCR platforms often allow the analysis of three to five pathogens in one run; however, since many viruses and bacteria are involved in BRD, these analyses can become extensive and expensive. Therefore, we previously developed and established a high-throughput RT-qPCR (ht-RT-qPCR) system for the detection of 11 respiratory and enteric viral and bacterial bovine pathogens, of which nine are related to respiratory disease [14]. The performance of the system has been tested on field samples (NS and fecal samples) from 100 Danish, intensive, commercial herds (83 dairy and 17 rosé-veal herds) [14]. The ht-RT-qPCR platform BioMark HD (Standard BioTools, South San Francisco, CA, USA) and the dynamic array (DA) integrated fluidic circuit (IFC) nanofluidic chip, which contains microfluidic networks that automatically combine samples and RT-qPCR reagents in reaction chambers, have been utilized. Using this ht-RT-qPCR system, it is possible to analyze samples for both viral and bacterial pathogens at a much lower cost than traditional techniques.

The objective of this study was to compare the pathogens detected by ht-RT-qPCR in NS and nBAL, investigate the agreement between NS and nBAL for different pathogens associated with respiratory disease in calves, and investigate associations between the presence of pathogens and the occurrence of clinical BRD.

## 2. Materials and Methods

### 2.1. Herds and Calves

Samples were collected from nine Danish dairy herds and three Danish fattening herds (rosé-veal calf) from September 2018 to February 2019. Each of the three fattening herds received calves from three of the included dairy herds. The data were a subsample of the data presented by Goecke et al. 2021 [14], where paired nasal swabs (NSs) and non-endoscopic bronchoalveolar lavage (nBAL) were collected from a subset of the calves. A total of 152 calves were enrolled in the present study. All calves were born in one of the nine dairy herds and followed up in two cohorts until approximately 3 months of age, depending on their production status as dairy heifers or fattening calves. Herds belonged to one of three clusters based on the receiving fattening herd, meaning that all dairy herds in a cluster delivered calves to the same fattening herd. To make it feasible to consider samples independent in the statistical analyses, repeated samples from the same individual were only included if sampled with more than a 45-day interval. The young dairy calves were primarily kept in single pens and in group pens when old, while all calves in the fattening herds were kept indoors in group pens. The feeding regimes differed according to local management. The distributions of calves sampled across age groups and herd types are given in Table 1.

Additionally, 203 of the 233 paired observations were combined with a clinical examination of the calves on the day of sampling. The clinical examination included observations of nasal and ocular discharge (0: none, 1: serous discharge, and 2: mucopurulent or purulent discharge or crusts), coughing (0: no cough, 1: single cough manually provoked, and 2: spontaneous coughing or repeated provoked coughs), and measurements of rectal temperature. Findings were registered electronically on a tablet on site. Based on the clinical observations, an overall disease score was created by assigning each calf to be either healthy or with clinical signs of respiratory disease (RespSick) on the day of sampling. Calves meeting one or more of the following criteria were considered RespSick:

1. Cough score 2 and nasal discharge score 2;
2. Cough score 2 and rectal temperature ≥ 39 °C;
3. Nasal discharge score 1 or 2 and rectal temperature ≥ 39 °C;
4. Nasal discharge score 1 or 2 and ocular discharge score 2.

### 2.2. Sample Collection

After the clinical examination, NSs were collected as follows: a 15 cm unguarded sterile cotton swab was placed three to seven cm into one nostril and rotated against the mucosal surface for a few seconds before withdrawal [15]. No prior cleaning of the nostril was performed. The tip of the swab was placed in a 5 mL Eppendorf tube containing 1.5 mL of phosphate-buffered saline. Then, the calves were lightly sedated with an intravenous administration of xylazine (0.06 mg/kg) and placed in sternal recumbency. The tip of the tongue was pulled out of the calf’s mouth to enable the visualization of the entrance to the trachea, where a calf flush catheter (proVETnordic, Ref.: 20096) was inserted to collect the nBAL sample. The catheter was placed in the trachea until resistance was felt by the veterinarian collecting the sample, 50 mL isotonic NaCl was flushed into the lungs, and as much fluid as possible was aspirated back into the syringe. The aspirated fluid was collected in 50 mL centrifuge tubes. Both NS and nBAL fluid samples were kept refrigerated (approximately 5 °C) for up to 48 h and, depending on the herd location, delivered or shipped in a box containing freezer packs to the Centre for Diagnostics at the Technical University of Denmark (DTU), where the samples were stored at −80 °C until nucleic acid extraction.

### 2.3. High-Throughput Real-Time PCR Analysis

Nucleic acids (RNA and DNA) were extracted from the NS and nBAL samples using the extraction robot QIAcube HT (QIAGEN, Hilden, Germany) and the IndiSpin QIAcube HT Pathogen Kit (Indical Bioscience, Leipzig, Germany) according to the manufacturer’s instructions. Prior to extraction, NS and nBAL samples were prepared by vortexing, followed by centrifugation for 5 min at 9000× *g*, and 200 µL of the supernatant was used for extraction. Positive and negative (nuclease-free water, Amresco, Cleveland, OH, USA) controls were included in each extraction. The nucleic acids were stored at −80 °C until further analysis.

Prior to the ht-RT-qPCR analysis, the extracted samples were run through a reverse transcription and/or pre-amplification step as described in Goecke et al. [14]. For the RT-qPCR analysis, the ht-RT-qPCR platform BioMark HD (Standard BioTools) and the BioMark 192.24 DA IFC chip (Standard BioTools) were used. For each sample, a 4 μL sample mix containing 2 μL of the TaqMan gene expression master mix (2×) (Applied Biosystems, Waltham, MA, USA), 0.2 μL of the sample loading reagent (20×) (Standard BioTools), and 1.8 μL of the preamplified sample were prepared. For each assay, a 4 μL of an assay mix containing 2 μL of an assay loading reagent (2×) (Standard BioTools) and 2 μL of primer–probe stock (final concentration: 16 μM primers and 5 μM probe) were prepared. A three (3)-microliter sample mix and 3 μL assay mix were loaded into the respective inlets of the 192.24 DA IFC chip. The 192.24 DA IFC chip was placed in the IFC controller RX (Standard BioTools) for loading and mixing for approximately 30 min and then subject to thermal cycling in the BioMark HD (Standard BioTools) with the following cycle conditions: 50 °C for 2 min, 95 °C for 10 min, followed by 40 cycles of 95 °C for 15 s and 60 °C for 60 s. Samples were tested in single reactions, and the assays were performed in duplicates. In each 192.24 DA IFC chip run, positive and negative (nuclease-free water) controls were included. Data, including Cq values and amplification curves, obtained on the BioMark system, were analyzed using the Fluidigm Real-Time PCR Analysis software version 4.5.2 (Standard BioTools) [14]. The used primer and probe sequences can be found in Supplementary Table S1. The NS and nBAL samples were analyzed for the presence of RNA and DNA material from nine bovine pathogens known to be able to cause respiratory disease: BCoV, BRSV, IDV, MH, PM, HS, M*spp.*, MB, and TP.

### 2.4. Data Analyses

All data management and analyses were conducted using R (version 4.3.1) [16]. Agreement between the PCR results from the two sample types was assessed by visualizing trends in patterns and the strength of the linear relationships between the cycle quantification (Cq) values of the included pathogens in nBAL and NS, using a heat map with the annotation of the correlation coefficient (rho). This was conducted by creating a correlation matrix within and between sample types in all calves, as well as within the two differentiated groups, young and old. Due to the nature of data, the non-parametric Spearman’s rho (ρ) was used.

To assess the associations of pathogens with respiratory disease, the test results were dichotomized according to the technical cut-off defining Cq values ≤ 25 as positive and Cq > 25 as negative. Significant differences between the occurrence of respiratory disease in calves with positive and negative test results for each pathogen were assessed using McNemar and chi-squared tests.

Finally, based on an initial univariable screening according to the strategy proposed by Hosmer and Lemeshow [17], a multivariable model was developed to assess combinations of the nine pathogens in NS and nBAL, together with the age group (young/old) and their effect on respiratory disease. The model was a generalized mixed linear model with herd type as a random factor. All analyses used a significance level of *p* = 0.05 to denote explanatory variables with a significant association with a RespSick calf.

## 3. Results

Descriptive data for the included samples and calves from the clusters and herds are presented in Table 2. Due to practical issues, no calves in the old category were sampled in herd 3.3. Still, seven calves originated in this herd were sampled as old in cluster 3.0. The mean age of the calves within the age group young was 13.4 days (SD 8.5), while the mean age amongst the old group was 82.3 days (SD 15.7). A total of 129 observations originated from heifer calves and 104 from bull calves.

### 3.1. Pathogen Patterns across Age Groups

**Figure 1:**
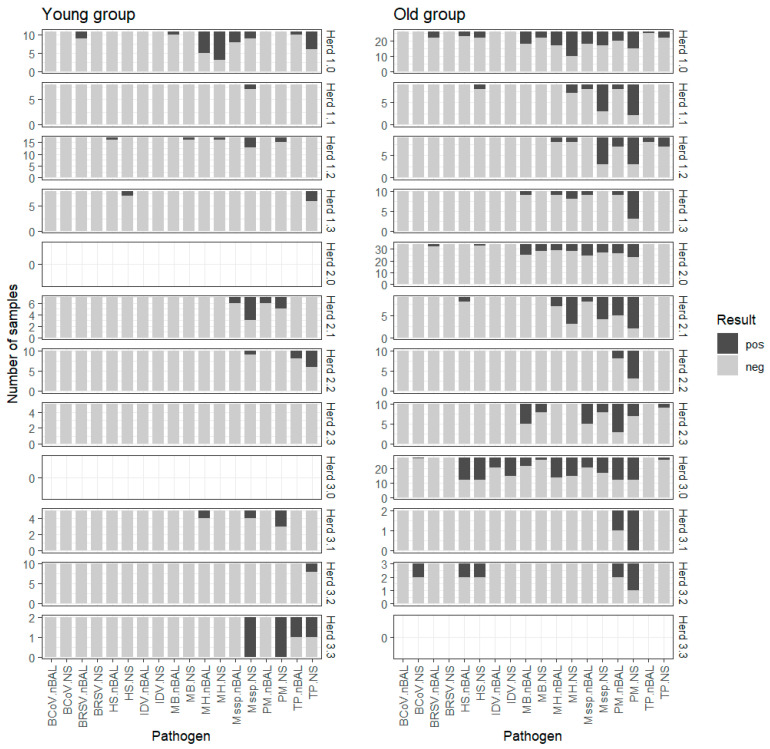
The distribution of positive (dark gray) and negative (gray) high-throughput real-time qPCR results for nine pathogens assessed in 233 paired non-endoscopic bronchoalveolar lavage (nBAL) and nasal swabs (NSs) collected from calves in 12 Danish cattle herds. The age groups are *young* (age < 35 days) and *old* (≥35 days). BcoV—bovine coronavirus, BRSV—bovine respiratory syncytial virus, HS—*Histophilus somni*, IDV—influenza D virus, MB—*Mycoplasma bovis*, MH—*Mannheimia Haemolytica* M*spp.—Mycoplasma* species, PM—*Pasteurella multocida*, TP—*Trueperella pyogenes*.

The distribution of pathogens in NS and nBAL samples among the two age groups are depicted in Figure 1, showing differences in patterns across herds and age groups within the herds. There were differences in pathogen patterns between young and old (Table 3). While low frequencies of positive pathogen results were found in NS and nBAL in the young calves, the old calves had higher frequencies across pathogens and sample types; however, the average number of old calves were numerically higher in the three fattening herds (Herds 1.0, 2.0, and 3.0). Interestingly, MB was more frequently detected in nBAL samples compared to NS samples in old calves, while M*spp.* dominated the positive samples primarily in NSs in young calves. Across the age groups the most frequently detected pathogens in both NS and nBAL were PM, M*spp.* and MH. In the young calves, it was M*spp.*, followed by TP and MH, while for the old calves, it was PM, followed by MH and M*spp.* (Table 3).

The viral pathogens BRSV, BCoV, and IDV showed herd-specific presence with higher frequency in the old calves. BCoV was only found in NSs in old calves in Herd 3.2, IDV was found in NSs and nBAL only in Herd 3.0, and BRSV was only found in nBAL in young and old calves (Figure 1).

### 3.2. Comparison of Single Pathogen Results in NS and nBAL

The results of the Spearman’s rank correlation coefficients (ρ) are given in Supplementary Table S2. Overall, the best correlation between single pathogen detection in both nBAL and NSs was found for MB in the young calves (ρ = 0.71) equal to a high correlation according to Hinkle et al. [18]; however, there was a low overall correlation (ρ = 0.33) due to the negligible correlation within the old calves (ρ = 0.26). HS showed moderate correlation between NS and nBAL in the old calves (ρ = 0.66) and overall (ρ = 0.64) while being negative for the young calves (ρ = −0.1). Low correlations for MH both in young calves (ρ = 0.56) and old calves (ρ = 0.45) resulted in an overall low correlation (ρ = 0.45). IDV was only present in old calves (and in one herd) at a moderate correlation (ρ = 0.61) between NS and nBAL.

### 3.3. Correlations between Different Pathogen Results

The strongest overall correlation between different pathogens was observed between MB and M*spp.* in nBAL with a Spearman’s rho of 0.72, driven by the high correlation in the old group (ρ = 0.74) despite the low correlation in the young group (ρ = 0.41). The second best correlations within the young group were found for combinations of PM and M*spp.* in NS (ρ = 0.44); TP in NS and MH in nBAL (ρ = 0.4); and finally, MH in NS and HS in nBAL (ρ = 0.34). Within the age group old, correlations appeared lower, with the best achieved between MB in NSs and M*spp.* in NSs (ρ = 0.4), IDV in NSs and MH nBAL (ρ = 0.34) and MH in NSs and HS in nBAL (ρ = 0.24).

### 3.4. Associations of Pathogens with Respiratory Disease

Statistically significant associations between RespSick and pathogens were only found for MH (Table 4). In young calves, this association was found for both nBAL (*p* < 0.001) and NS (*p* = 0.002), while for the old calves, it was only found for nBAL (*p* = 0.02). IDV showed a tendency to be associated with signs of respiratory disease in old calves (*p* = 0.1), but this should be interpreted with caution, as IDV was only present in one herd and with a small sample size.

More than half (54%) of the 104 RespSick calves tested positive for one to three pathogens simultaneously, 31.6% (31) for four–eight pathogens, while 16.3% (n = 17) had negative pathogen results (Table 5). Amongst the healthy calves, 58.2% (n = 57) tested positive for at least one pathogen. There were statistically significant more healthy calves with no pathogens detected but no statistically significant differences between the number of positive pathogens and being RespSick (Table 5).

Based on the univariable screening, 10 pathogens with a *p* < 0.2 constituted the factors in the initial logistic regression model associating the probability of having signs of respiratory disease:

RespSick ~ BRSV nBAL + MH nBAL *+* HS nBAL *+* PM nBAL + MB NS + M*spp.* NS + MH NS + HS NS + IDV NS + PM NS + AgeGroup

The backwards selection process resulted in only two pathogens being included in the final model: RespSick ~ BRSV nBAL + MH nBAL + AgeGroup (Table 6). Being a young calf significantly lowered the risk of having respiratory disease, while a positive test for MH in nBAL significantly increased the risk of having respiratory disease.

## 4. Discussion

This study addresses multiple age-related differences in respiratory disease and pathogen occurrence in NS and nBAL samples tested with a ht-RT-qPCR in bovine calves sampled in Danish dairy and veal cattle farms. The highest detection frequencies of both disease and pathogens were in calves that were aged 35 days or older. Poor to moderate agreement was found between the NS and nBAL results. The multivariable statistical model results showed that being less than 35 days old lowered the risk of having clinical BRD, while the presence of MH nBAL was associated with clinical BRD. Also, a potential link was found between BRD and IDV in older calves, although it was only found in one herd in a smaller subsample of calves/sample size.

We found PM to be the most prevalent pathogen detected among Danish calves, both in NS (38.4%) and nBAL (23.8%). This is driven by its presence in calves > 35 days of age and were detected in especially NS. In Poland and Belgium, PM has also been found to be one of the most prevalent pathogen detected from dairy calves in relation to BRD [19,20]. DeRosa et al. [9] found that 70% of the NS cultures of PM and MH was genetically identical to the corresponding bacteria found in the lungs by nBAL. In their study, the positive predictive value for NS to correctly detect a PM-positive sample in TTA was 100%, while the negative predictive value was 67% [21] and a strong to moderate agreement between PM in NS and TTA was found [8]. Despite being the most prevalent pathogen, PM was not significantly associated with respiratory disease in either young or old calves in our study (Table 4), and PM did not stay in the final multivariable model of associations between pathogens and respiratory disease in calves (Table 4). This fits well with PM being a secondary pathogen as part of the ubiquitous resident flora [5].

MH was the second most detected pathogen, both in NS and nBAL. This high prevalence agrees with other studies [19,20]. The moderate correlation of 0.56 between MH in NS and nBAL for the young age groups (Table S2) suggests that both NS and nBAL can be used to detect if MH is present in the airways of a calf. In support of this, Doyle et al. [8] found very good agreement between MH detected by NS and TTA.

The detection of MH in nBAL was the only sample–pathogen combination that was associated with respiratory disease in both young and old calves, and MH was also associated with respiratory disease in young calves when detected in NS (Table 4). MH is described as a secondary pathogen but with the potential to be a primary pathogen in connection with virulence factors and other risk factors [6]. This is supported by our study, as we found MH and BRSV in nBAL were the only variables included in the final model predicting the risk of BRD (Table 6). The log-odds of being RespSick increased by 1.87 with positive MH in nBAL (Table 6).

BRSV in nBAL was also included in the final model, which supports the understanding of BRSV as a primary pathogen, capable of inducing disease without bacterial infections present [6]. We did not find any BRSV-positive NS and only eight nBAL samples were positive. The agreement between the two methods is therefore non-existing in our data, which is in line with other authors, who have found moderate agreement between NS and TTA [8]. This suggests that samples from the lower airways are superior to NSs for BRSV detection.

MB and M*spp.* were the third most detected pathogen group, primarily driven by many positive samples from the old calves, in both NS and nBAL. Only one sample was positive for MB among the YOUNG calves, and in general, there were few diseased young calves. MB and M*spp.* are also found to be a prevalent BRD pathogens in other European countries [19,20]. A weak correlation (0.33) between MB findings in NS and nBAL and the overrepresentation of nBAL findings (Table S2) suggest that for the individual calf, nBAL is superior to NSs in detecting MB. This is supported by Thomas et al. [22] and Pohjanvirta et al. [10]; the latter found that NS was the most cost-effective if looking for MB at the herd level, while sampling the lower airways was recommended for detection in diseased animals.

Neither MB nor M*spp.* were statistically significantly associated with respiratory disease in young or old calves (Table 4). This is in line with the perception that the pathogenicity of MB and M*spp.* spans widely, i.e., from commensal to opportunistic and primary pathogens [6,23]. This unclear causality makes it difficult to interpret and diagnose mycoplasmas in relation to respiratory disease and suggests that the synergy effect of MB is not associated with specific pathogens. Despite MB often being found in combination with other pathogens, no strong correlations (Table S2) with other pathogens were found in our study. However, the strongest overall correlation between different pathogens was observed between MB and M*spp.*, which makes good sense since M*spp.* assay covers the known bovine Mycoplasma species including MB. 

As an interesting case, IDV was detected for the first time in Denmark during sampling for the overarching *Robust Calves project* [24] which the present study also was related to. The presence of IDV in Herd 3.0 was more prominent in NS (13 positive) compared to nBAL (7 positive), which was not surprising, since the clinical symptoms in the IDV positive calves were dominated by a nasal discharge score of 2 and simultaneously an itchy conjunctivitis given by an ocular discharge score of 2. Nine of the thirteen (69%) IDV-positive calves were classified as RespSick and were also positive for MH, HS, and PM at the same time. This picture of IDV aiding opportunistic secondary bacterial infections is in line with previous findings [3,25] may support the importance of viral surveillance, especially in fattening herds.

BRD is a multifactorial disease, and pathogens, hosts, and environmental factors play important roles in which animals develop disease [2]. We only found that MH and BRSV were associated with respiratory disease and our data might show a tendency of RespSick calves having more pathogens detected than healthy calves, but none were statistically significant (Table 5). This is consistent with the broad acceptance that healthy calves can harbor the same pathogens as calves with BRD. In our study, less of the young calves were RespSick compared to the old calves, making this comparison tricky, as the old calves were primarily from fattening herds, where the pathogen loads were higher and the mixing of calves form different herds more likely than in the dairy herds. This aids in the discussion of how difficult it is to design and interpret diagnostic tests for BRD [6].

When designing diagnostic test guidelines for a disease complex as BRD, there are several considerations to consider, e.g., which animals to sample, what material to sample and which pathogens to look for. The results from our study emphasize that there is no one-fits-all solution when deciding which techniques and material to sample. Knowledge of which pathogens are expected is a minimum requirement to be able to design a test protocol since you are only able to detect the target(s) the test is specific for. However, the use of the ht-RT-qPCR system enables the detection of the pathogens of interest since the samples are tested for several targets within the same test. Nonetheless, positive PCR findings do not necessarily equal clinical disease since some pathogens are commensal inhabitants of the respiratory tract. In contrast to bacterial culture, PCR detects the mere presence of pathogen DNA, and there might be a longer window where the pathogen can be detected despite not being responsible for clinical disease. Furthermore, RT-qPCR is a very sensitive method that is able to detect pathogens at low concentrations. In a previous study, a correlation between disease and pathogen load has been investigated and pathogen specific cut-offs for some pathogens in NS have been established [26], while to the author’s knowledge, no clinical cut-offs for nBAL have been published. Therefore, the present study used the analytical cut-off based on a Cq-value of 25. Further investigations of the clinical cut-offs in nBAL should be pursued for practical application of the testing under field conditions. The purpose of testing is also important, as decisions on test materials can be dependent on the purpose of sampling, e.g., control program (like monitoring of pathogens at the herd level) vs. the diagnosis of individual calves [10].

This study was a cross-sectional study, where we had either one sample per calf or at least 45 days between the samples to consider them independent. This means that the timely association between pathogens and disease could not be measured. Determining disease state based on clinical signs is not straightforward, and despite showing the same clinical signs, the calves are likely to be at different stages of the disease course. We can therefore not determine if the association between disease and pathogen would have been clearer on a specific day in the disease course. The different pathogens probably have different predilection sites in the calves, but it could also be that the association between NSs and nBAL was time dependent, e.g., that early in the disease course NS are positive and nBAL becoming positive in the later stages of disease. This is not possible to investigate in our study due to the cross-sectional nature of the data.

## 5. Conclusions

Across all pathogens, the agreement between NSs and nBAL were at the most moderate, indicating that NSs are relatively poor predictors of pathogens in the lower respiratory tract. The highest detection frequencies of both disease and pathogens were in calves that were aged 35 days or older. Only the presence of MH in the NS and nBAL in younger calves, and in the nBAL in older calves, was associated with clinical BRD. Despite the wide presence of MB and M*spp.*, none of these were statistically significantly associated with respiratory disease. In conclusion, the present study confirms the complexity of pathogen detection in BRD, with marked influences of age and sampling method on pathogen detection and disease associations.

## Figures and Tables

**Table 1:** Distribution of samples taken from 152 calves collected in nine dairy and three fattening herds across two age groups, *young* (age < 35 days) and *old* (≥35 days), either once, twice or exclusively in the given age group.

Sex	Herd Type	Young (Once)	Young (Twice)	Young (Exclusively)	Old (Once)	Old (Twice)	Old (Exclusively)	Young + Old
Heifer	Dairy	63	0	17	59	1	13	48
	Fattening	0	0	0	5	0	5	
Bull	Dairy	0	0	9	0	0	1	18
	Fattening	11	0	1	57	13	0	
	Total	74	0	27	121	14	19	66

**Table 2:** Overview of 233 observations from calves in the two age groups, *young* (age < 35 days) and *old* (≥35 days), collected in 12 Danish cattle herds. To illustrate which herds were linked together in clusters, the number before the decimal separator denotes the cluster number, and the number after the decimal separator denotes the herd number within the cluster.

Cluster and Herd	Herd Type	Observations	Young	Median Age (Min–Max)	Old	Median Age (Min–Max)
1.0	Fattening	37	11	23 (22–33)	26	83 (40–123)
1.1	Dairy	17	8	13 (4–29)	9	76 (59–91)
1.2	Dairy	26	17	13 (2–33)	9	76 (41–88)
1.3	Dairy	18	8	8.5 (6–14)	10	78.5 (72–84)
2.0	Fattening	34	0	-	34	86.5 (42–96)
2.1	Dairy	16	7	22 (18–27)	9	82 (74–89)
2.2	Dairy	20	10	5 (3–7)	10	82 (80–84)
2.3	Dairy	15	5	4 (1–4)	10	81 (78–87)
3.0	Fattening	28	0	-	28	87.5 (73–113)
3.1	Dairy	7	5	11 (5–25)	2	77 (74–80)
3.2	Dairy	13	10	11 (4–17)	3	75 (72–78)
3.3	Dairy	2	2	11 (11)	0	-
Total		233	83		150	

**Table 3:** Distribution of positive test results from the high-throughput real-time qPCR in two age groups *young* (age < 35 days) and *old* (≥35 days) among 233 samples collected using non-endoscopic bronchoalveolar lavage (nBAL) and nasal swabs (NSs) from 152 Danish calves. Both indicate the number of paired observations that tested positive in both sample types.

				Positive Test Results					
	All			Young Calves			Old Calves		
Pathogen	nBAL	NS	Both	nBAL	NS	Both	nBAL	NS	Both
BRSV	8	0	0	2	0	0	6	0	0
BCoV	0	2	0	0	2	0	0	0	0
MB	30	15	9	1	1	0	29	14	9
M*spp.*	37	61	14	4	15	1	33	46	13
MH	39	55	31	7	9	5	32	46	22
HS	22	24	16	1	1	0	21	23	16
IDV	7	6	6	0	0	0	7	6	6
PM	50	87	32	1	8	0	49	79	32
TP	6	23	5	4	14	4	2	9	1

BRSV—bovine respiratory syncytial virus, BCoV—bovine coronavirus, MB—*Mycoplasma bovis*, M*spp.*—*Mycoplasma* species, MH—*Mannheimia Haemolytica*, HS—*Histophilus somni*, IDV—influenza D virus, PM—*Pasteurella multocida*, TP—*Trueperella pyogenes*.

**Table 4:** Distribution of positive test results from non-endoscopic bronchoalveolar lavage (nBAL) and nasal swabs (NSs) assessed by the high-throughput real-time qPCR across age groups *young* (age < 35 days) and *old* (≥35 days) assessed for calves with signs of respiratory disease (RespSick) and without clinical signs (healthy) among 203 samples with clinical registrations collected from 152 Danish calves.

Pathogen	Total Samples (%)	Young Calves		Old Calves	
		RespSick	Healthy	RespSick	Healthy
	N = 203	N = 13/60	N = 47/60	N = 91/143	N = 52/143
nBAL					
BRSV	8 (4.0)	2 (3.3) ^†^	0	5 (3.5)	2 (1.4)
BCoV	0	0	0	0	0
MB	29 (14.4)	0	1 (1.7)	17 (12.0)	11 (7.7)
M*spp.*	37 (18.3)	1 (1.7)	3 (5.0)	20 (14.1)	13 (9.1)
MH	38 (18.8)	6 (10.0) ***	1 (1.7)	26 (18.3) *	5 (3.5)
HS	20 (9.9)	0	1 (1.7)	5 (3.5)	14 (9.9)
IDV	7 (3.4)	0	0	7 (4.9) ^†^	0
PM	48 (23.8)	0	1 (1.7)	33 (23.2)	14 (9.9)
TP	5 (2.0)	1(1.7)	2 (3.3)	1 (0.7)	1 (0.7)
NS					
BRSV	0	0	0	0	0
BCoV	2 (1.0)	0	0	2 (1.4)	0
MB	14 (6.9)	0	1 (1.7)	10 (7.1)	4 (2.8)
M*spp.*	46 (22.7)	2 (3.3)	5 (8.3)	29 (20.4)	10 (7.1)
MH	51 (25.2)	6 (10.0) **	1 (1.7)	30 (21.1)	12 (8.5)
HS	22 (10.8)	0	1 (1.7)	16 (11.3)	6 (4.2)
IDV	13 (6.4)	0	0	9 (6.3)	4 (2.8)
PM	78 (38.4)	0	1 (1.7)	50 (35.2)	24 (16.9)
TP	21 (10.4)	5 (8.3)	7 (11.7)	5 (3.5)	4 (2.8)

Chi-squared test significance: ^†^ *p* < 0.1, * *p* < 0.05, ** *p* < 0.01, *** *p* < 0.001. B BRSV— bovine respiratory syncytial virus, BCoV—bovine coronavirus, MB—*Mycoplasma bovis*, M*spp.*—*Mycoplasma* species, MH—*Mannheimia Haemolytica*, HS—*Histophilus somni*, IDV—influenza D virus, PM—*Pasteurella multocida*, TP—*Trueperella pyogenes*.

**Table 5:** Distribution of positive pathogens detected by the high-throughput real-time PCR in both non-endoscopic bronchoalveolar lavage (nBAL) and nasal swab (NS) samples from 152 Danish calves. *p*-value is given for testing the difference between the numbers of positive pathogens and being RespSick.

Positive Tests	Healthy	RespSick	Total	*p*-Value
0	41	17	58	<0.001 ***
1	29	27	56	0.68
2	10	16	26	0.37
3	10	13	23	0.77
4	4	12	16	0.09 ^†^
5	2	7	9	0.2
6	1	5	6	0.24
7	0	5	5	0.08 ^†^
8	0	2	2	0.5
9	1	0	1	0.98
Total	98	104	202	

Chi-squared test significance: ^†^ *p* < 0.1, *** *p* < 0.001.

**Table 6:** Parameter estimates for the prediction of showing clinical signs of respiratory disease in 203 cases among Danish calves.

Coefficient		Estimate (S.E.)	*p*-Value
Intercept		0.230 (0.19)	0.23
BRSV nBAL	Positive	1.916 (1.17)	0.10
MH nBAL	Positive	1.872 (0.51)	0.0002
Age Group	Young	−1.899 (0.39)	<0.001

BRSV—bovine respiratory syncytial virus, MH—*Mannheimia Haemolytica*.

## Data Availability

The raw data supporting the conclusions of this article will be made available by the authors on request.

## Supplementary Materials

The following supporting information can be downloaded at: https://www.mdpi.com/article/10.3390/pathogens13060479/s1, Table S1: Primer and probe sequences used for detection of viruses and bacteria [12,14,27,28,29,30,31,32]; Table S2: Correlation coefficients given by Spearman’s ρ between nine different pathogens assessed in non-endoscopic bronchoalveolar lavage (nBAL) and nasal swabs (NSs) collected from calves within their first three month of life in 12 Danish cattle herds.
