# Supplementary material for: Comparing Occurrence of Bovine Respiratory Pathogens Detected by High-Throughput Real-Time PCR in Nasal Swabs and Non-Endoscopic Bronchoalveolar Lavage Samples from Dairy and Veal Calves
Source: Pathogens. 2024 Jun 5;13(6):479. doi: 10.3390/pathogens13060479 (PMC11206406; doi:10.3390/pathogens13060479)
Supplement: Supplementary file 1 [file pathogens-13-00479-s001.zip › Supplementary table 1_Primer and probe sequences.pdf]

Supplementary Table 1. Primer and probe sequences used for detection of viruses and bacteria.

Pathogen	Target gene	Name	Sequence (5'-3')	Length (bp)	Reference
Bovine respiratory syncytial virus	F	BRSV-F-485F<br>BRSV-F-569R<br>BRSV F Taqman-546	AAGGGTCAAACATCTGCTTAACTAG<br>TCTGCCTGWGGGAAAAAAG<br>FAM-AGAGCCTGCATTRTCACAATACCACCCA-BHQ1	85	[1]
Bovine coronavirus	M	BCoV-F<br>BCoV-R<br>BCoV-P	GTTGGTGGAGTTTCAACCCAG<br>GGTAGTCCTCAATTATCGGCC<br>FAM-CATCCTTCCCTTCATATCTATACACATC-BHQ1	90	F, R and P (modified):<br>[2]
H. somni	16S rRNA	HS-F<br>HS-R<br>HS-P	GAAGATACTGACGCTCGAGT<br>TTCGGGCACCAAGTRTTCA<br>FAM-TCCCCAAATCGACATCGTTTACAGCGTG-BHQ1	115	F and P: [3]<br>R : [4]
Influenza D virus	PB1	Inf D-F<br>Inf D-R<br>Inf D-P	GCTGTTTGCAAGTTGATGGG<br>TGAAAGCAGGTAACTCCAAGG<br>FAM-TTCAGGCAAGCACCCGTAGGATT-BHQ1	136	[5]
M. haemolytica	*sodA*	M. hae-F<br>M. hae-R<br>M. hae-P	GCCGTTGTTTCAACCGCTAAC<br>CGTGTTCCCAAACGTCTAAGAC<br>FAM-TCGGATAGCCTGAAACGCCTGCCAC-BHQ1	100	[3]
M. bovis	*oppD*	PMB996-F<br>PMB1066-R<br>Mbovis1016	TCAAGGAACCCCACCAGAT<br>AGGCAAAGTCATTTCTAGGTGCAA<br>FAM-TGGCAAACTTACCTATCGGTGACCCT-TAMRA	71	[6]
Mycoplasma spp.	16S rRNA	Mycoplasma-F<br>Mycoplasma-R<br>Mycoplasma-P	GATCCTGGCTCAGGATGAAC<br>CGTTGAGTACGTGTTACTCAC<br>FAM-GGCTGTGTGCCTAATACATGCATGTCG-BHQ1	103	[3]
P. multocida	*kmt1*	PM-ny-F<br>PM-ny-R<br>PM-P	GACTACCGACAAGCCCACTC<br>CTATCCGCTATTTACCCAGTGG<br>FAM-GTGCGAATGAACCGATTGCCGCG- BHQ1	125	F and R: [3]<br>P: [7]
T. pyogenes	plo-Pyolysin	T. pyogenes-F<br>T. pyogenes-R<br>T. pyogenes-P	**C**ATCAACAATCCCACGAAGAG<br>TTGCAGCATGGTCAGGATAC<br>FAM-CCGTGACTCAAGGACTGAACGGCCT-BHQ1	98	F (modified) and R from [8]<br>P: [3]

References:

1. Hakhverdyan, M. ; Hägglund, S; Larsen, L.E. ; Belák, S. Evaluation of a single-tube fluorogenic RT-PCR assay for detection of bovine respiratory syncytial virus in clinical samples. *J Virol Methods.* 2005,123(2):195-202. doi: 10.1016/j.jviromet.2004.09.016.

2. Decaro, N. ; Elia,G. ; Campolo, M. ; Desario, C. ; Mari, V. ; Radogna, A. ; Colaianni, M.L. ; Cirone, F. ; Tempesta, M. ; Buonavoglia, C. Detection of bovine coronavirus using a TaqMan-based real-time RT-PCR assay. *J Virol Methods*. 2008,151(2):167-171. doi: 10.1016/j.jviromet.2008.05.016.

3. Goecke, N.B.; Nielsen, B.H.; Petersen, M.B.; Larsen, L.E. Design of a High-Throughput Real-Time PCR System for Detection of Bovine Respiratory and Enteric Pathogens. *Front Vet Sci* 2021, *8*, doi:10.3389/fvets.2021.677993.

4. Angen, Ø.; Ahrens, P.; Tegtmeier, C. Development of a PCR test for identification of *Haemophilus somnus* in pure and mixed cultures. *Veterinary Microbiology*, 1998, 63(1):39-48, https://doi.org/10.1016/S0378-1135(98)00222-3.

5. Hause, B.M. ; Ducatez, M. ; Collin, E.A.; Ran, Z.; Liu, R.; Sheng, Z.; Armien, A.; Kaplan, B.; Chakravarty, S.; Hoppe, A.D.; Webby, R.J.; Simonson, R.R.; Li, F. Isolation of a novel swine influenza virus from Oklahoma in 2011 which is distantly related to human influenza C viruses. *PLoS Pathog*. 2013,9(2):e1003176. doi: 10.1371/journal.ppat.1003176.

6. Sachse, K.; Salam, H.S.; Diller, R.; Schubert, E.; Hoffmann, B.; Hotzel, H. Use of a novel real-time PCR technique to monitor and quantitate Mycoplasma bovis infection in cattle herds with mastitis and respiratory disease. *Vet J*. 2010, 186(3):299-303. doi: 10.1016/j.tvjl.2009.10.008.

7. Goecke, N.B.; Hjulsager, C.K.; Krog, J.S.; Skovgaard, K.; Larsen, L.E. Development of a high-throughput real-time PCR system for detection of enzootic pathogens in pigs. *J Vet Diagn Invest*. 2020, 32(1):51-64. doi: 10.1177/1040638719890863.

8. Kishimoto, M.; Tsuchiaka, S.; Rahpaya, S.S.; Hasebe, A.; Otsu, K.; Sugimura, S.; Kobayashi, S.; Komatsu, N.; Nagai, M.; Omatsu, T.; Naoi, Y.; Sano, K.; Okazaki-Terashima, S.; Oba, M.; Katayama, Y.; Sato, R.; Asai, T.; Mizutani, T. Development of a one-run real-time PCR detection system for pathogens associated with bovine respiratory disease complex. *J Vet Med Sci*. 2017, 18;79(3):517-523. doi: 10.1292/jvms.16-0489.
