# Supplementary material for: Comparing Occurrence of Bovine Respiratory Pathogens Detected by High-Throughput Real-Time PCR in Nasal Swabs and Non-Endoscopic Bronchoalveolar Lavage Samples from Dairy and Veal Calves
Source: Pathogens. 2024 Jun 5;13(6):479. doi: 10.3390/pathogens13060479 (PMC11206406; doi:10.3390/pathogens13060479)
Supplement: Supplementary file 1 [file pathogens-13-00479-s001.zip › Supplemetary Table 2_Spearmans rho.pdf]

Supplementary Table 2. Correlation coefficients given by Spearman´s ϱ between nine different pathogens assessed in non-endoscopic bronchoalveolar lavage (nBAL) and nasal swabs (NS) collected from calves within their first three month of life in 12 Danish cattle herds.

All		nBAL									NS								
		BRSV	BCoV	MB	Mspp	MH	HS	IDV	PM	TP	BRSV	BCoV	MB	Mspp	MH	HS	IDV	PM	TP
nBAL	BRSV	1																	
	BCoV	NA	1																
	MB	0.16	NA	1															
	Mspp	0.07	NA	0.72	1														
	MH	0.1	NA	0.22	0.21	1													
	HS	-0.06	NA	0.11	0.12	0.23	1												
	IDV	-0.04	NA	0	-0.02	0.23	0.19	1											
	PM	-0.05	NA	0.13	0.06	0.13	0.19	0.16	1										
	TP	-0.03	NA	0.01	0.06	0.07	-0.05	-0.03	-0.09	1									
NS	BRSV	NA	NA	NA	NA	NA	NA	NA	NA	NA	1								
	BCoV	-0.02	NA	-0.04	-0.04	-0.04	-0.03	-0.02	0.04	-0.02	NA								
	MB	0.25	NA	0.33	0.24	0.15	0.11	-0.05	0.05	-0.05	NA								
	Mspp	0.19	NA	0.12	0.12	0.16	0.1	0	0.05	0.12	NA	0.05	0.38						
	MH	0.19	NA	0.12	0.16	0.45	0.27	0.15	0.02	0.02	NA	-0.05	0.13	0.22					
	HS	-0.07	NA	0.12	0.09	0.28	0.64	0.27	0.15	0.03	NA	0.12	0.05	0.13	0.25	1			
	IDV	-0.05	NA	0	0.04	0.32	0.3	0.61	0.19	-0.04	NA	-0.02	0.13	-0.04	0.19	0.29	1		
	PM	-0.09	NA	-0.01	-0.05	0.06	0.15	0.15	0.28	-0.03	NA	-0.01	0.11	0.17	0.12	0.16	0.22	1	
	TP	0.08	NA	0.02	-0.04	0.24	-0.06	-0.06	-0.09	0.29	NA	-0.03	0.1	0	0.11	-0.07	0.05	-0.08	1

Young		nBAL									NS								
		BRSV	BCoV	MB	Mspp	MH	HS	IDV	PM	TP	BRSV	BCoV	MB	Mspp	MH	HS	IDV	PM	TP
nBAL	BRSV	1																	
	BCoV	NA	1																
	MB	-0.02	NA	1															

	Mspp	-0.04	NA	0.41	1														
	MH	0.25	NA	-0.03	0.09	1													
	HS	-0.02	NA	-0.01	-0.03	-0.03	1												
	IDV	NA	NA	NA	NA	NA	NA	1											
	PM	-0.03	NA	-0.02	0.22	-0.06	-0.02	NA	1										
	TP	-0.04	NA	-0.02	0.18	0.12	-0.02	NA	-0.04	1									
NS	BRSV	NA	NA	NA	NA	NA	NA	NA	NA	NA	1								
	BCoV	NA	NA	NA	NA	NA	NA	NA	NA	NA	NA	1							
	MB	-0.02	NA	0.71	0.28	-0.05	-0.02	NA	-0.03	-0.04	NA	NA	1						
	Mspp	-0.07	NA	-0.05	0.16	-0.03	0.19	NA	0.07	0.18	NA	NA	0.15	1					
	MH	0.2	NA	-0.04	0.23	0.56	0.34	NA	-0.07	0.1	NA	NA	-0.05	0.15	1				
	HS	-0.02	NA	-0.01	-0.03	-0.03	-0.01	NA	-0.02	-0.02	NA	NA	-0.02	-0.05	-0.04	1			
	IDV	NA	NA	NA	NA	NA	NA	NA	NA	NA	NA	NA	NA	NA	NA	NA	1		
	PM	-0.05	NA	-0.04	-0.08	-0.1	0.31	NA	0.16	0.12	NA	NA	-0.05	0.44	0.01	-0.04	NA	1	
	TP	0.11	NA	-0.05	0.02	0.4	-0.05	NA	-0.09	0.32	NA	NA	-0.07	-0.06	0.31	-0.05	NA	-0.06	1

Old		nBAL									NS								
		BRSV	BCoV	MB	Mspp	MH	HS	IDV	PM	TP	BRSV	BCoV	MB	Mspp	MH	HS	IDV	PM	TP
nBAL	BRSV	1																	
	BCoV	NA	1																
	MB	0.16	NA	1															
	Mspp	0.07	NA	0.74	1														
	MH	0.05	NA	0.22	0.22	1													
	HS	-0.09	NA	0.04	0.09	0.23	1												
	IDV	-0.05	NA	-0.05	-0.05	0.23	0.16	1											
	PM	-0.09	NA	0.03	-0.05	0.1	0.13	0.14	1										
	TP	-0.03	NA	0.07	0.06	0.11	-0.05	-0.03	-0.08	1									
NS	BRSV	NA	NA	NA	NA	NA	NA	NA	NA	NA	1	NA							
	BCoV	-0.03	NA	-0.06	-0.06	-0.06	-0.05	-0.03	0.01	-0.01	NA	1							

MB	0.29	NA	0.26	0.19	0.17	0.09	-0.08	-0.01	-0.04	NA	-0.04	1						
Mspp	0.27	NA	0.09	0.07	0.18	0.05	-0.03	-0.03	0.15	NA	0.05	0.4	1					
MH	0.16	NA	0.06	0.09	0.4	0.24	0.14	-0.07	0.03	NA	-0.08	0.12	0.18	1				
HS	-0.09	NA	0.06	0.05	0.29	0.66	0.25	0.07	0.09	NA	0.11	0.02	0.11	0.25	1			
IDV	-0.07	NA	-0.06	0	0.34	0.28	0.6	0.16	-0.04	NA	-0.04	0.11	-0.08	0.17	0.28	1		
PM	-0.16	NA	-0.15	-0.17	0	0.04	0.12	0.14	-0.04	NA	-0.06	0.06	0.03	0.02	0.07	0.17	1	
TP	0.08	NA	0.14	-0.02	0.24	-0.01	-0.06	-0.02	0.2	NA	-0.03	0.25	0.11	0.1	-0.03	0.13	0.03	1

BRSV - bovine respiratory syncytial virus, BCoV- bovine coronavirus, MB - *Mycoplasma bovis, Mspp - Mycoplasma* species, MH – *Mannheimia Haemolytica,* HS - *Histophilus somni*, IDV - influenza D virus, PM - *Pasteurella multocida, TP - Trueperella pyogenes.*
